# Supplementary figures and images for: Genome-Wide Investigation of BAM Gene Family in Annona atemoya: Evolution and Expression Network Profiles during Fruit Ripening
Source: Int J Mol Sci. 2023 Jun 22;24(13):10516. doi: 10.3390/ijms241310516 (PMC10341951; doi:10.3390/ijms241310516)

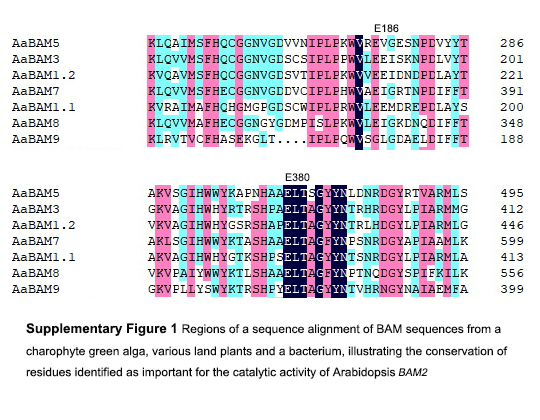

Supplement: Supplementary file 1 [file ijms-24-10516-s001.zip › Figure S1.jpg]
